# Presence of *Listeria monocytogenes* in Mediterranean-Style Dry Fermented Sausages

**DOI:** 10.3390/foods4010034

**Published:** 2015-03-12

**Authors:** Domenico Meloni

**Affiliations:** Department of Veterinary Medicine, University of Sassari, Via Vienna 2, 07100, Sassari, Italy; E-Mail: dmeloni@uniss.it; Tel.: +0039-079-229-570; Fax: +0039-079-229-458

**Keywords:** *Listeria monocytogenes*, Mediterranean area, dry fermented sausages, hurdle technology

## Abstract

The morphological, physiological and epidemiological features of *L. monocytogenes*, together with the severity of human listeriosis infections, make *L. monocytogenes* of particular concern for manufacturers of cold-stored “ready to eat” (RTE) foods. *L. monocytogenes* has been isolated from a wide variety of RTE foods and is responsible for several outbreaks associated with the consumption of RTE meat, poultry, dairy, fish and vegetable products. Although *L. monocytogenes* is among the most frequently-detected pathogens in dry fermented sausages, these products could be included in the category of RTE products in which the growth of *L. monocytogenes* is not favored and have rarely been implicated in listeriosis outbreaks. However, *L. monocytogenes* is highly difficult to control in fermented sausage processing environments due to its high tolerance to low pH and high salt concentration. In many Mediterranean-style dry fermented sausages, an empirical application of the hurdle technology often occurs and the frequent detection of *L. monocytogenes* in these products at the end of ripening highlights the need for food business operators to properly apply hurdle technology and to control the contamination routes of *L. monocytogenes* in the processing plants. In the following, through an up-to-date review of (personal and un-) published data, the main aspects of the presence of *L. monocytogenes* in Mediterranean-style dry fermented sausages will be discussed.

## 1. Introduction to the Main Features of *Listeria monocytogenes*

### 1.1. Taxonomy

The genus Listeria comprises fifteen species, *i.e*., *L. monocytogenes*, *L. ivanovii*, *L. innocua*, *L. welshimeri*, *L. seeligeri*, *L. grayi*, *L. marthii*, *L. rocourtiae*, *L. leichmannii*, *L. weihenstephanensis*, *L. floridensis*, *L. aquatic*, *L. cornellensis*, *L. riparia* and *L. grandensis* (Meloni, 2014 [1]).

### 1.2. Morphology of Listeria monocytogenes

Members of the genus *Listeria* are non-spore-forming, facultative anaerobic and small Gram-positive rods (0.5–4 µm in diameter and 0.5–2 µm in length). Peritrichous flagella give them a typical tumbling motility, occurring at 20–25 °C. Based on somatic (O) and flagellar (H) antigens, 13 serotypes of *L. monocytogenes* have been recognized. These are identified alphanumerically: 1/2a, 1/2b, 1/2c, 3a, 3b, 3c, 4a, 4ab, 4b, 4c, 4d, 4e and 7 (Meloni, 2014 [1]). Serotypes 1/2a, 1/2b and 1/2c are the most frequently isolated from food or the food production environment.

### 1.3. Physiology of Listeria monocytogenes

*L. monocytogenes* is catalase-positive, oxidase-negative and is able to survive between 0 and 45 °C. The optimum growth temperature is around 30–37 °C. *L. monocytogenes* can grow at pH ranges between 4.5 and 9.0 (optimum pH between 6 and 8) and is able to multiply in food matrices at water activity (a_w_) values of 0.92 and in NaCl concentrations of 12%, generally lethal to other microorganisms. *L. monocytogenes* is a ubiquitous organism, widely distributed in the environment: the principal reservoirs are soil, forage and water (Sauders and Wiedmann, 2007 [2]; Todd and Notermans, 2011 [3]; European Food Safety Authority (EFSA), 2014 [4]). Other reservoirs include healthy humans and animals (International Life Sciences Instistute (ILSI), 2005 [5]) or infected domestic and wild animals (EFSA, 2014 [4]). *L. monocytogenes* is a psychrotrophic bacterium, can multiply at low temperatures, both under aerobic and anaerobic conditions, adapt to disinfectants and adhere to various surfaces (Arevalos-Sánchez *et al*., 2012 [6]). *L. monocytogenes* is widespread in food processing facilities and has been isolated from different processing environments. Once introduced into the processing plants, it is able to survive and persist for a long time under adverse conditions (Farber and Peterkin, 1991 [7]; Gram *et al.*, 2007 [8]; Gandhi and Chikindas, 2007 [9]). The biofilm forming ability is an important cause for such persistence (Cruz and Fletcher; 2012 [10]; Fonnesbech Vogel *et al.*, 2001 [11]). In the pork meat supply chain, *L. monocytogenes* has been repeatedly isolated (Nesbakken *et al.*, 1996 [12]), with an increase of contamination along the production line (Chasseignaux *et al.*, 2002 [13]).

## 2. *Listeria monocytogenes* as a Foodborne Pathogen

### 2.1. Pathogenicity of Listeria monocytogenes

*L. monocytogenes* is the etiologic agent of listeriosis. Human cases of listeriosis are almost exclusively caused by *L. monocytogenes*. Very rare cases of infections are attributed to *L. ivanovii* and *L. seeligeri.* The difference in the pathogenic potential of *L. monocytogenes* strains has been demonstrated by means of *in vivo* bioassay and *in vitro* cell assay (Soni *et al.*, 2014 [14]). Whereas some *L. monocytogenes* strains are naturally virulent, inflicting high morbidity and mortality, others are non-virulent and unable to infect the mammalian host (Liu *et al.*, 2003 [15]; Velge and Roche, 2010 [16]). The discrimination between pathogenic and non-pathogenic strains is imperative to assess the possible significance of this microorganism from food safety and public health aspects (Jensen *et al.*, 2008 [17]; Roberts *et al.*, 2009 [18]). Rasmussen *et al.* (1995 [19]) and Wiedmann *et al.* (1997 [20]) demonstrated that molecular typing methods can also allow *L. monocytogenes* to divide into three evolutionary lineages characterized by different pathogenic potentials: Lineage I, strains associated with epidemic outbreaks of listeriosis (serotypes 1/2b, 3b, 4b, 4d and 4e); Lineage II, strains isolated from sporadic cases of listeriosis (serotypes 1/2a, 1/2c, 3a and 3c); Lineage III, strains rarely associated with cases of listeriosis (serotypes 4a and 4c) (Wiedmann, 2002 [21]). The lineage status of serotypes 4ab and 7 still remains unclear due to limited availability of such strains (World Organization for Animal Health (OIE), 2014 [22]). The majority of the infections caused by *L. monocytogenes* are thought to be food-borne, and infections most often affect the central nervous system, the bloodstream and the pregnant uterus. Two forms of listeriosis have been described in humans, and symptoms vary, ranging from febrile gastroenteritis in healthy people (Piana *et al.*, 2005 [23]), to life-threatening invasive infections characterized by septicemia and meningoencephalitis in risk groups, such as young, old, pregnant and immune-compromised (YOPI) people (De Cesare *et al.*, 2007 [24]).

### 2.2. Epidemiology of Listeriosis

Listeriosis is an important disease in Europe: it is the fourth most common zoonotic disease, and it has an annual incidence of 0.41 cases per 100,000 population, with the highest notification rates in Finland, Spain and Denmark (EFSA, 2014 [4]). In 2012, 1642 confirmed human cases were reported, mostly domestically acquired. A statistically significant increasing trend was observed over the period 2008–2012, with a +10.5% increase compared with 2011 (EFSA, 2014 [4]). As reported by previous authors, the highest notification rates were reported in persons aged 65 years and above (Denny and McLauchlin, 2008 [25]) and in persons aged below one year. Transmission during pregnancy was highlighted in 79% of the cases reported in newborns, with the spread of the infection to the fetus which is born severely ill. Listeriosis has the highest hospitalization rate cases of all zoonoses under EU surveillance: 91.6% of the cases with supplementary long-term sequelae. Listeriosis is the third leading cause of death in the EU after West Nile fever and trichinellosis and the first among food-borne pathogens, with an estimated case fatality rate of 17.8% (EFSA, 2014 [4]). A total of 198 deaths were reported in 2012, the highest number of fatal cases reported since 2006 (EFSA, 2014 [4]). The morphological, physiological and epidemiological features of *L. monocytogenes*, together with the severity of human listeriosis infections, make *L. monocytogenes* of particular concern for manufacturers of cold-stored “ready to eat” (RTE) foods (Romanova *et al.*, 2002 [26]; Van Coillie *et al.*, 2004 [27]; Shen *et al.*, 2006 [28]). *L. monocytogenes* has been isolated from a wide variety of RTE foods and is responsible for several outbreaks associated with the consumption of RTE meat, poultry, dairy, fish and vegetable products (Aureli *et al.*, 2000 [29]; Gillespie *et al.*, 2006 [30]; Public Health Agency Canada (PHAC), 2009 [31]; U.S. Department of Agriculture’s Food Safety and Inspection Service (USDA/FSIS), 2010 [32]; Todd and Notermans, 2011 [3]). The EU legislation (Regulation (EC) No. 2073/2005 [33]) lays down food safety criteria for *L. monocytogenes* in RTE foods, and it is generally considered that concentrations of *L. monocytogenes* greater than 100 CFU/g are required to cause human disease in healthy populations. Qualitative results alone are not necessarily an indicator of public health risk. In RTE products intended for infants and for special medical purposes, *L. monocytogenes* must be absent in 25 g, while in RTE products in which the growth of *L. monocytogenes* is not favored (pH ≤ 4.4 or a_w_ ≤ 0.92 or pH ≤ 5.0 and a_w_ ≤ 0.94), *L. monocytogenes* must not be present at levels exceeding 100 CFU/g during the shelf-life. In RTE foods able to support its growth, *L. monocytogenes* must be absent in 25 g at the time of leaving the production plant. However, if the producer can demonstrate that the product will not exceed the limit of 100 CFU/g throughout its shelf-life, this criterion does not apply (European Commission, 2005 [33]). In 2012 the highest reported levels of non-compliance in RTE samples taken at processing were observed in fishery products (8.0%) and unspecified cheeses (3.4%). The highest proportions of food samples exceeding the legal safety limit, at retail, were observed in fishery products (0.5%) and fermented meat sausages (0.4%) (EFSA, 2014 [4]). Pork meat products, e.g., fermented sausages that are contaminated by *L. monocytogenes* at more than 100 CFU/g and that are to be consumed without further heat treatment, are considered to form a direct risk to human health.

## 3. *Listeria monocytogenes* in the Pork Meat Processing Industry

### 3.1. Swine Slaughterhouses

*L. monocytogenes* has been previously found in every stage along the pork processing industry (Thévenot *et al.*, 2006 [34]; López *et al.*, 2008 [35]), including swine slaughterhouses (Sammarco *et al.*, 1997 [36]; Korsak *et al.*, 1998 [37]). The sources of *L. monocytogenes* contamination during swine slaughtering are pig-related and environmental (Bonardi *et al.*, 2002 [38]). *L. monocytogenes* is spread to the carcass mainly from the carrier animal: the pathogen has been occasionally isolated from feces and from the skin of healthy carriers with intestinal colonization (Autio *et al.*, 2000 [39]).

#### 3.1.1. Prevalence of *Listeria monocytogenes*

The prevalence in feces is generally comprised between 0% and 50% (Belœil *et al.*, 2003 [40]). This wide range of prevalence is probably due to the emptying of the rectum before evisceration, an operation that usually helps to reduce the extent of dissemination and the consequent fecal contamination of carcasses (Kanuganti *et al.*, 2002 [41]). The role of live animals as a source of processing environment contamination and, consequently, pork carcasses has been demonstrated: the most contaminated areas are usually represented by the area of stunning/hanging (Gobat and Jemmi, 1991 [42]; Nesbakken *et al.*, 1994 [43]; Saide-Albornoz *et al.*, 1995 [44]; Borch *et al.*, 1996 [45]). Contamination may occur during the evisceration, because of the breaking of the intestine (Adesyiun *et al.*, 1995 [46]), and reaches a prevalence of around 60%–65% (Thévenot *et al.*, 2005 [47]; López *et al.*, 2008 [35]), highlighting the profound influence of good hygiene practices and equipment cleanliness on carcass contamination (Bonardi *et al.*, 2002 [38]; Meloni *et al.*, 2013 [48]). Several authors (Buncic *et al.*, 1991 [49]; Ripamonti *et al.*, 2002 [50]; Kanuganti *et al.*, 2002 [41]; Autio *et al.*, 2003 [51]; Fabbi *et al.*, 2005 [52]) correlated the contamination of equipment and consequently of the carcasses with the presence of the pathogen in other niches, such as the tongue (14%) and tonsils (7%–61%). This wide range of prevalence is probably due to differences in sampling techniques and/or methods of farm management.

#### 3.1.2. Serotypes of *Listeria monocytogenes*

The most frequent serotypes found in carcasses and slaughterhouse environments are 1/2a, 1/2c (Hof and Rocourt, 1992 [53]; Thévenot *et al.*, 2005 [47]; Meloni *et al.*, 2013 [48]), while serotype 1/2b is generally found at low prevalence rates.

### 3.2. Meat Processing Plants

The level of *L. monocytogenes* contamination tends to increase along the pork supply chain (López *et al.*, 2008 [35]). Raw meat is an important source of contamination of working environments and equipment. The most contaminated zones are the areas of the receipt of raw materials, the cells of refrigeration and the processing rooms (Chasseignaux *et al.*, 2002 [13]).

#### 3.2.1. Prevalence of *Listeria monocytogenes*

A higher prevalence is found in raw meat (45%–50%) compared to the muscles of freshly slaughtered pigs (0%–2%). Raw meat represents the primary source of contamination of final products by *L. monocytogenes* (Giovannacci *et al.*, 1999 [54]; Kathariou, 2002 [55]; Kanuganti *et al.*, 2002 [41]; Thévenot *et al.*, 2005 [47]). In turn, due to the presence of favorable conditions for growth and multiplication during the processing stages of cooling and cutting, the prevalence of *L. monocytogenes* in minced meat intended to be processed ranges between 16% and 50% (Jay, 1996 [56]; Chasseignaux *et al.*, 2002 [13]). The level of contamination increases significantly up to 70%–100% during the processing stages of grinding and bagging (Nesbakken *et al.*, 1996 [12]; Thévenot *et al.*, 2005 [47]). Pork meat can also be cross-contaminated through contact with work surfaces and equipment. The level of contamination of the surfaces in contact and without contact with meat during processing ranges between 17%–50% and 11%–25%, respectively (Thévenot *et al.*, 2005 [47]; Mureddu *et al.*, 2014 [57]).

#### 3.2.2. Persistence of *Listeria monocytogenes*

Once introduced into the plants, *L. monocytogenes* can persist over time in the processing environment (López *et al.*, 2008 [35]), forming assemblages of surface-associated microbial cells enclosed in hydrated extracellular polymeric substances and growing in biofilms (Mafu *et al.*, 1990 [58]; Gandhi and Chikindas, 2007 [9]; Meloni *et al.*, 2012 [59]). In recent surveys carried out in fermented sausage processing plants located in Italy (Meloni *et al.*, 2012 [59]; Meloni *et al.*, 2014 [60]; Mureddu *et al.*, 2014 [57]), the evaluation of the *in vitro* biofilm production of *L. monocytogenes* strains isolated from several surfaces in contact and without contact with meat showed a low short-time persistence (3–4 months) capacity and weak or moderate ability to form biofilm after 24 to 40 h of incubation. Isolates from serotypes 1/2a, 1/2b and 4b presented higher adherence when compared to isolates from serotype 1/2c (Meloni *et al.*, 2014 [60]). Harborage sites, such as meat grinders, work tables or floor drains, can be critical sites for the processing plant environment (Tompkin, 2002 [61]). This could be due to the common presence of meat in these environmental niches: this would likely produce a common food-conditioning film, which might select for adhesion, growth and biofilm formation by isolates with a common prophage type (Verghese *et al.*, 2011 [62]). Decontaminating surfaces in contact and without contact with meat is especially challenging, because, when entrapped in a biofilm, *L. monocytogenes* is afforded unusual protection against available disinfectants and treatments (Zhao *et al.*, 2004 [63]). Without suitable sanitization procedures, the presence of *L. monocytogenes* increases the food safety risk (Samelis and Metaxopoulos, 1999 [64]; Meloni *et al.*, 2014 [60]). Previous authors have reported that the recommended concentrations of commercial sanitizers are higher than required (Cruz *et al.*, 2012 [10]). In a recent survey (Mureddu *et al.*, 2014 [57]), the *in vitro* evaluation of the resistance to disinfectants (chlorine substances and quaternary ammonium compounds, both at 37% concentration) showed a reduction of *L. monocytogenes* growth after 24, 48 and 72 h of incubation in isolates from processing environments and finished products.

#### 3.2.3. Serotypes of *Listeria monocytogenes*

Several studies have shown that strains of *L. monocytogenes* isolated from meat-processing environments belong mainly to serotypes 1/2c and 1/2a (Chasseignaux *et al.*, 2002 [13]; Thévenot *et al.*, 2006 [34]; Mureddu *et al.*, 2014 [57]; Meloni *et al.*, 2014 [60]).

## 4. Production of Mediterranean-Style Dry Fermented Sausages

Mediterranean-style dry fermented sausages are characterized by their relatively longer shelf-life and the exceptional hygienic background, which is brought about by the production of lactic acid in the fermentation process (pH < 4.5–5) and low water activity (<0.90) of the final product (Ordóñez and de la Hoz, 2007 [65]). In general, in the manufacturing of fermented sausages, meat and fat involves selection, chopping and mincing and mixing with curing ingredients, spices and authorized additives. At the end of the ripening and drying process, they come out as cured meat products (Ordóñez and de la Hoz, 2007 [65]). Traditionally, fermented sausages are made using lactic acid bacteria (LAB) and Gram-positive catalase positive cocci, in particular coagulase-negative staphylococci (CNS) naturally present in the meat or with the inoculation of starter cultures at the chopping step. The mixture is then filled in natural or artificial casings, left to ferment and then dried. In the Mediterranean area, regional customs, environmental variations and family recipes have given rise to a wide range of fermented sausages, and it can be said that there are almost as many types of sausages as there are geographical regions or even manufacturers, although their production process always requires the combination of fermentation and dehydration (Ordóñez and de la Hoz, 2007 [65]). The very wide range of Mediterranean dry fermented sausages can be classified according to a range of criteria (Table 1), such as the acidity, the mincing size of meat and fat, the addition or absence of molds on the surface, the addition of ingredients and the diameter and type of casing used (Ordóñez and de la Hoz, 2007 [65]).

### 4.1. Preliminary Stages

The meat used depends on eating habits, customs and the preferences prevailing in the geographical region where the fermented sausage is produced (Table 1). This is usually pork, sometimes mixed with beef (Ordóñez and de la Hoz, 2007 [65]). The fat should be firm, with a high melting point and a low content of polyunsaturated fatty acids, because this causes the fermented sausage to turn rancid more quickly (Frey, 1985 [66]). Mincing of the meat and fat is done at low temperatures (between −5 and 0 °C) to achieve a clean cut and to avoid the release of intramuscular fat from fatty meats, which could cause changes in the color and the drying process during ripening (Frey, 1985 [66]). Once the meat and fat have been comminuted, the starter culture (LAB) and the nitrate reducing CNS, curing salts, additives (nitrates, nitrites, glutamate) and other ingredients (sugars, aromatic herbs and spices) are added. The mixture, after refrigerated storage overnight, is placed in a kneader and stuffed into natural or synthetic casings (Greco *et al.*, 2005 [67]; Ordóñez and de la Hoz, 2007 [65]). The sausages are then ripened.

### 4.2. Fermentation and Ripening

After filling and the first warming up at 20–22 °C for 4–6 h, the fermentation stage for the manufacture of a standard dry fermented sausage can be summarized as follows: one to two days at 18–24 °C and 60% relative humidity (RH) and five days at 15 °C and 70% RH (Ordóñez and de la Hoz, 2007 [65]). After fermentation, ripening is carried out for five to 15 days in store rooms at 15 °C and 70%–75% RH. These conditions are maintained until the end of the ripening period, during which many flavor compounds develop (Ordóñez and de la Hoz, 2007 [65]). The normal pH of the majority of Mediterranean-style fermented sausages is close to 4.5/5.4, which has several beneficial effects on both the manufacturing process and the shelf-life (Greco *et al.*, 2005 [67]; Ordóñez and de la Hoz, 2007 [65]). However, in some low acid fermented sausages (e.g., Soudjouk, Fuet), the final pH is close to 6.0/6.7. The suppression of the acid hurdle can compromise the safety of these products (Ordóñez and de la Hoz, 2007 [65]; Jofré *et al.*, 2009 [68]). At the end of ripening, the water activity of fermented sausages is close to 0.90, which inhibits bacterial growth. The water activity hurdle is strengthened with time and is largely responsible for the stability of fermented sausages (Ordóñez and de la Hoz, 2007 [65]).

### 4.3. Hurdle Technology in Mediterranean-Style Dry Fermented Sausages

*L. monocytogenes* is inhibited in fermented sausages by sequential steps: the “hurdle technology” concept includes several sequential hurdles, essential at different stages of the fermentation or ripening process (Barbuti and Parolari, 2002 [69]). Due to the sequence of these hurdles, pathogenic and spoilage bacteria are effectively inhibited in Mediterranean-style dry fermented sausages, and the desired competitive flora (especially LAB) is selected (Leistner, 1995 [70]). These hurdles are essential in different steps of the fermentation or ripening process and lead to stable and safe final products (Leistner and Gould, 2002 [71]). In the early steps of the fermentation process, nitrite and salt added together in the form of nitrite-curing salts inhibit many bacteria in the initial product, such as pseudomonads and other Gram-negative oxidative organisms, which rapidly multiply and spoil uncured meats in the presence of oxygen (Leistner and Gould, 2002 [71]). Other bacteria, such as CNS, are able to multiply, use up the oxygen and cause the decrease of the redox potential of the product to decrease. CNS are also important, because of other biochemical-metabolic properties, such as lipolytic activity. Together with the nitrate reduction, these affect the quality (color and flavor) and the stability of the products (Selgas *et al.*, 1994 [72]; Greco *et al.*, 1999 [73]; Mazzette *et al.*, 1999 [74]). This, in turn, favors the redox potential hurdle, which inhibits aerobic bacteria and promotes the selection of LAB (Leistner and Gould, 2002 [71]). This competitive microflora flourish by metabolizing the added sugars, producing lactic acid, bacteriocins and inhibitory metabolites, causing a decrease in pH value and an increase of the pH hurdle (Papa, *et al.*, 1993 [75]; Torriani *et al.*, 1994 [76]; Grazia *et al.*, 1998 [77]; Leroy and de Vuyst, 1999 [78]; Hebert *et al.*, 2000 [79]; Lucke, 2000 [80]). These properties explain why LAB are important as starter cultures in the manufacturing of dry fermented sausages (Greco *et al.*, 2005 [67]). This is of particular importance for the microbial stability of quick-ripened fermented sausages, which are not greatly dried. In long-ripened fermented sausages, nitrite is depleted, and lactic acid bacteria slowly die. On the contrary, the redox potential and pH increase again (Leistner and Gould, 2002 [71]). Only the water activity hurdle is strengthened with time, and this hurdle is then largely responsible for the stability of long-ripened sausages (Leistner, 1987 [81]). This sequence of hurdles inhibits pathogenic and spoilage bacteria inside Mediterranean-style dry fermented sausages, whereas undesirable mold growth on the surface of the sausages is inhibited by smoke or by the use of desirable mold starter cultures (Leistner and Gould, 2002 [71]).

**Table 1:** Formulation (g/100 g) of typical Mediterranean-style dry fermented sausages *.

Formulation	Salchichón/Saucisson (Spain/France) (1)	Salami (Italy) (1)	Salsiccia Sarda (Italy) (2)	Fuet (Spain) (1)	Chorizo (Spain/Portugal) (1)	Lukanka (Bulgaria) (3)
Lean pork	35/70	45–84	85–87	60–70	65–80	25
Pork fat	10–25	14–25	5–8	30–40	20–40	20
Lean beef	0–50	0–37	-	0–20	0–20	55
Sugars	0.2–0.5	0.3–0.7	0.8	0.1–0.4	0.6–0.8	-
Curing salts	2.0–2.4	1.8–2.5	3	2.0–2.4	1.8–2.1	2.24
Whole/ground black pepper	0/0.2–0.2/0.4	0/0.2–0.1/0.14	0.25	0/0.2–0/0.3	-	0.30
White pepper	-	0–0.2	0.8	-	0–0.3	-
Paprika	-	-	-	-	0/1.5–1.5/2.5	-
Red pepper	-	-	-	-	-	0.20
Cumin	-	-	-	-	-	0.20
Garlic	-	0–0.2	0.15	-	0.2–1.2	-
Sodium Glutamate	0.25	-	-	0–0.15	-	-
Powdered milk	0–0.6	0–2.5	-	-	0–2.5	-
Caseinate	0–0.6	-	-	0–1.0	-	-
Liquid smoke	-	-	-	-	-	0.20

* Adapted and modified from Ordóñez and de la Hoz, 2007 [65]*.* Sources: (1) Ordóñez and de la Hoz, 2007 [65]; (2) Meloni *et al.*, 2012 [59]; (3) Balev *et al.*, 2005 [82].

## 5. *Listeria monocytogenes* in Mediterranean-Style Dry Fermented Sausages

### 5.1. Prevalence of Listeria monocytogenes

Fermented meat products may be contaminated by *L. monocytogenes* at several stages. The raw materials may be contaminated from the slaughterhouse environment, during the production process or by contact with contaminated unprocessed raw materials, unclean surfaces or people (Chasseignaux *et al.*, 2002 [13]; Thévenot *et al.*, 2006 [34]) in the post-processing stages (Colak *et al.*, 2007 [83]). *L. monocytogenes* is among the most frequently-detected pathogens in dry fermented sausages, and several studies have documented the prevalence of the pathogen in fermented sausages (Table 2), reaching prevalence levels of up to 40%–45% (Cantoni *et al.*, 1989 [84]; Cordano and Rocourt, 2001 [85]; Levine *et al.*, 2001 [86]; Thévenot *et al.*, 2005 [47]; De Cesare *et al.*, 2007 [24]; Meloni *et al.*, 2009 [87]; Meloni *et al.*, 2012 [59]; Mureddu *et al.*, 2014 [57]; Meloni *et al.*, 2014 [60]; Doménech *et al.*, 2015 [88]).

### 5.2. Levels of Contamination by Listeria monocytogenes

Fermented sausages contaminated with *L. monocytogenes* have rarely been implicated in critical listeriosis outbreaks (EFSA, 2014 [4]). Fermented sausages have moderate rates of consumption and serving sizes in many countries. The risk per serving is low (2.1 × 10^−12^), and the global number of annual cases per 100,000 people is only 0.0000055 (USDA/FSIS, 2010 [32]). Many Mediterranean-style dry fermented sausages could be included in the category of RTE products in which the growth of *L. monocytogenes* is not favored, although there is great variability depending on the local traditions that influence fermentation and ripening (Hospital *et al.*, 2012 [89]). Mostly in the manufacturing of traditional fermented sausages marketed locally or regionally, an empirical application of the hurdle technology often occurs. Some manufacturers tend to reduce the ripening period in order to increase profitability. As a matter of fact, the pH and aw of these products are often within the limits for growth of *L. monocytogenes* (Hospital *et al.*, 2012 [89]). Insufficiently dried sausages may have water activity levels close to 0.92–0.94 (Meloni *et al.*, 2014 [60]), and *L. monocytogenes* is able to survive during sausage fermentation, overcoming the hurdles encountered during the manufacturing process. In general, the contamination levels at the end of ripening are always lower than 100 CFU/g (Farber and Peterkin, 1991 [7]), because *L. monocytogenes* cannot compete with the prevailing lactic acid bacteria. Only without competitive microflora *L. monocytogenes* is able to multiply and reach high levels of contamination (higher than 1000 CFU/g), representing a major public health concern (McLauchlin *et al.*, 2004 [90]; Thévenot *et al.*, 2006 [34]).

### 5.3. Serotypes of Listeria monocytogenes

As already noted for raw meat and meat-processing environments, also in the Mediterranean-style sausages at the end of ripening, serotypes 1/2c, 1/2a and 1/2b are more often detected (Jay, 1996 [56]; Thévenot *et al.*, 2006 [34]; Meloni *et al.*, 2014 [60]; Mureddu *et al.*, 2014 [57]), while serotype 4b is more rarely seen (Greenwood *et al.*, 1991 [91]; Hayes *et al.*, 1991 [92]). In Italy, serotype 1/2a is increasing its importance in the epidemiology of listeriosis. An increase of cases due to serotype 1/2a and a decline in cases due to serotype 4b have been reported by several authors (Gianfranceschi *et al.*, 2009 [93]; Pontello *et al.*, 2012 [94]). Despite the low prevalence rates of serotype 4b, previous surveys have shown that Lineage I strains of serotype 4b belonging to a clonal group (DUP-ID 1038) linked to several listeriosis outbreaks (De Cesare *et al.*, 2007 [24]) were recently recovered in Mediterranean-style dry fermented sausages produced in Italy (Meloni *et al.*, 2009 [87]).

**Table 2:** Prevalence of *Listeria monocytogenes* in naturally-contaminated Mediterranean-style dry fermented sausages *.

Type of Mediterranean-style dry fermented sausage	Prevalence%	Concentration data	pH of the final product	a_w_ of the final product
Fermented sausage **	10	<3 CFU/g	4.7–5.4	0.78–0.90
Italian salami **	13.3	Presence in 25 g	4.8–5.2	0.85–0.90
Soudjouk **	7	Presence in 25 g	4.9–6.7	*nd*
Fermented sausage **	3.25	Presence in 25 g	*nd*	*nd*
Fermented sausage **	20	Presence in 25 g	*nd*	*nd*
Fermented sausage **	19.05	Presence in 25 g	*nd*	*nd*
Fermented sausage **	44	Presence in 25 g	*nd*	*nd*
Fermented sausage **	20	Presence in 25 g	*nd*	*nd*
Salami **	16.67	Presence in 25 g	*nd*	*nd*
Salsiccia **	11.54	Presence in 25 g	*nd*	*nd*
Fermented sausage **	20	Presence in 25 g	*nd*	*nd*
Salami **	10	Presence in 25 g	*nd*	*nd*
Salami **	16	Presence in 25 g	*nd*	*nd*
Salami **	5	20 CFU/g	*nd*	*nd*
Fermented sausage **	10	Presence in 25 g	*nd*	*nd*
Salami **	40	Presence in 25 g	*nd*	*nd*
Spanish-style sausage **	3.70	Presence in 25 g	*nd*	*nd*
Salsiccia Sarda ***	20	Presence in 25 g	5.32	0.90
Salsiccia Sarda ****	8	Presence in 25 g	5.37	0.91

* Adapted and modified from Skandaminis and Nychas, 2007 [95]. *nd:* no data*.* Based on: ** Skandaminis and Nychas, 2007 [95]; *** Meloni *et al.*, 2009 [87]; **** Meloni *et al.*, 2014 [60].

## 6. Conclusions

The outcome of the previous paragraphs can be summarized from a safety standpoint as follows: Mediterranean-style fermented sausages may be contaminated with *L. monocytogenes* from various sources, including raw meat, slaughterhouse environments, production processes and post-processing conditions. In order to prevent these contamination sources, good manufacturing practices, correct sampling schemes, adequate cleaning and disinfection procedures and HACCP principles have to be applied. The use of starter cultures and the correct drying to lower the water activity can minimize the potential for growth of *L. monocytogenes* in Mediterranean-style fermented sausages. However, the frequent detection of *L. monocytogenes* at the end of ripening of these products highlights the need for food business operators to apply hurdle technology properly and to control the contamination routes of *L. monocytogenes* in meat processing plants.
